# Genome-Wide Identification and Expression Patterns of Cucumber Invertases and Their Inhibitor Genes

**DOI:** 10.3390/ijms241713421

**Published:** 2023-08-30

**Authors:** Chenze Qi, Liyun Xv, Wenhao Xia, Yunyi Zhu, Yudan Wang, Zhiping Zhang, Haibo Dai, Minmin Miao

**Affiliations:** 1 College of Horticulture and Landscape Architecture, Yangzhou University, Yangzhou 225009, China; 201804313@stu.yzu.edu.cn (C.Q.); 13338812015@163.com (L.X.); xiapanda0115@163.com (W.X.); wenzhe667@163.com (Y.Z.); dx120210135@stu.yzu.edu.cn (Y.W.); zhangzp@yzu.edu.cn (Z.Z.); dx120180088@yzu.edu.cn (H.D.); 2 Joint International Research Laboratory of Agriculture and Agri-Product Safety, Ministry of Education of China, Yangzhou University, Yangzhou 225009, China; 3 Key Laboratory of Plant Functional Genomics, The Ministry of Education, Jiangsu Key Laboratory of Crop Genomics and Molecular Breeding, Yangzhou University, Yangzhou 225009, China

**Keywords:** cucumber, invertase, invertase inhibitors, sucrose, gene family analysis

## Abstract

Invertases and their inhibitors play important roles in sucrose metabolism, growth and development, signal transduction, and biotic and abiotic stress tolerance in many plant species. However, in cucumber, both the gene members and functions of invertase and its inhibitor families remain largely unclear. In this study, in comparison with the orthologues of *Citrullus lanatus* (watermelon), *Cucumis melo* (melon), and *Arabidopsis thaliana* (Arabidopsis), 12 invertase genes and 12 invertase inhibitor genes were identified from the genome of *Cucumis sativus* (cucumber). Among them, the 12 invertase genes were classified as 4 cell wall invertases, 6 cytoplasmic invertases, and 2 vacuolar invertases. Most invertase genes were conserved in cucumber, melon, and watermelon, with several duplicate genes in melon and watermelon. Transcriptome analysis distinguished these genes into various expression patterns, which included genes *CsaV3_2G025540* and *CsaV3_2G007220*, which were significantly expressed in different tissues, organs, and development stages, and genes *CsaV3_7G034730* and *CsaV3_5G005910*, which might be involved in biotic and abiotic stress. Six genes were further validated in cucumber based on quantitative real-time PCR (qRT–PCR), and three of them showed consistent expression patterns as revealed in the transcriptome. These results provide important information for further studies on the physiological functions of cucumber invertases (*CSINVs*) and their inhibitors (*CSINHs*).

## 1. Introduction

Sucrose widely occurs in many higher plants [1], and many of its functions have been clarified. First, it is the main product of photosynthesis and serves as a carbon source for plant growth and development [2]. Second, it can be cleaved into glucose and fructose, providing energy for plant growth [3]. Third, in sink organs of crops, sucrose can accumulate and directly determine quality and production [4]. Fourth, as a small molecule, sucrose participates in osmotic regulation (accumulation under stress, regulating osmotic pressure) and can improve the stress resistance of plants [5]. Fifth, as a signaling molecule, sucrose regulates various developmental and metabolic processes, such as carbohydrate metabolism, the accumulation of storage proteins, sucrose transport, anthocyanin accumulation, floral induction [6], and leaf senescence [7]. The sucrose content of plant tissues depends on the balance between synthesis and decomposition, where invertase is one key enzyme catalyzing the destruction of sucrose [8,9].

According to its pH optimum and isoelectric point, invertase (EC 3.2.1.26) can be divided into acid invertase and alkaline/neutral invertase. Acid invertase is located in the cell wall or vacuole, while neutral invertase is in the cytoplasm [10]. Acid invertase has several essential functions, including regulating phloem sugar unloading, controlling sugar composition in storage organs, and modulating plant growth. For example, Yan et al. found that the cell wall invertase *MeCWINV3* regulates sugar distribution from source to sink and maintains sugar balance, thus affecting the yield of cassava [11]. Moreover, cell wall invertase catalyzes the hydrolysis of sucrose, and the entry of hexose into cells could serve as a signal to regulate cell division and differentiation [12]. Association analysis showed that the sorghum vacuolar invertase gene *SbVIN1* is related to stem traits such as stem length, stem thickness, internode number, stem fresh weight, and grain traits as well as 100-grain weight and grain width [13]. Alkaline/neutral invertase plays important roles in plant growth and development, starch synthesis, and other plant growth activities. In *Arabidopsis thaliana* (Arabidopsis), overexpression of the alkaline/neutral invertase gene *MeNINV1* resulted in higher invertase activity and increased glucose, fructose, and starch content in the leaves, which promoted plant growth and delayed flowering time, but no change was observed in its resistance to abiotic stress [14]. Similarly, overexpression of the cytoplasmic invertase gene *SoCIN1* in tobacco also increased the soluble sugar content and promoted plant growth [15].

The activity of invertase is regulated at the post-translational level via compartment-specific inhibitory proteins, referred to as invertase inhibitors. Invertase inhibitors belong to the pectin methylesterase inhibitor (PMEI) family, which has almost identical structural properties [16,17,18]. Invertase inhibitors can be classified into NtCIF (cell wall inhibitor of β-fructosidase) and NtVIF (vacuolar inhibitor of invertase). The NtCIF protein showed a broad range of activities against different plant cell wall invertases and vacuolar invertases, and the NtVIF protein was rather specific for vacuolar invertases [19]. In potato, both cell wall/apoplastic and vacuolar isoforms of inhibitors play an important role in resistance to cold-induced sweetening, which causes a great loss to the potato processing industry [20,21].

*Cucumis sativus* L. (cucumber) is an important vegetable crop that is widely cultivated. In contrast to other sucrose-transport-type plants, raffinose family oligosaccharides (RFOs) are one of the major translocated sugars in the vascular bundle of cucumber, which indicates that the functions of sucrose and invertase in cucumber may be different from those in many higher plants. Although stachyose is the main transport load of assimilates in cucumber, there is still a considerable concentration of sucrose in the phloem sap [22,23]. In addition, sucrose is an important carbohydrate that has a major impact on cucumber fruit quality [24]. Moreover, it is a signalling molecule that can regulate the growth and development of cucumber, such as pollen tube growth [25]. A recent report found that cucumber vacuolar invertase *CsVI2* could regulate sucrose metabolism and enhance drought stress in seedlings [26]. This indicates that invertase and its inhibitor genes also play important roles in cucumber. However, both the members and their functions of the two gene families remain largely unknown in cucumber.

To clarify the gene families of cucumber invertase (*CSINVs*) and its inhibitors (*CSINHs*) and identify critical members in regulating cucumber growth and development and biotic and abiotic stress tolerances, the reported genes of invertase and its inhibitors in melon, watermelon and Arabidopsis were used for blasting the genome of cucumber, and the number, physical and chemical properties of the orthologous genes in cucumber were identified combined with protein motifs, phylogeny, and in silico transcriptome analysis. Partial genes were further validated by quantitative real-time PCR (qRT–PCR). These results provide a genome-wide understanding of invertases and their inhibitor genes in cucumber, which will benefit further studies on the physiological functions of cucumber invertases (*CSINVs*) and their inhibitors (*CSINHs*).

## 2. Results

### 2.1. Identification, Motif Composition, and Gene Structural Analysis of CSINVs and CSINHs

**Figure 1:**
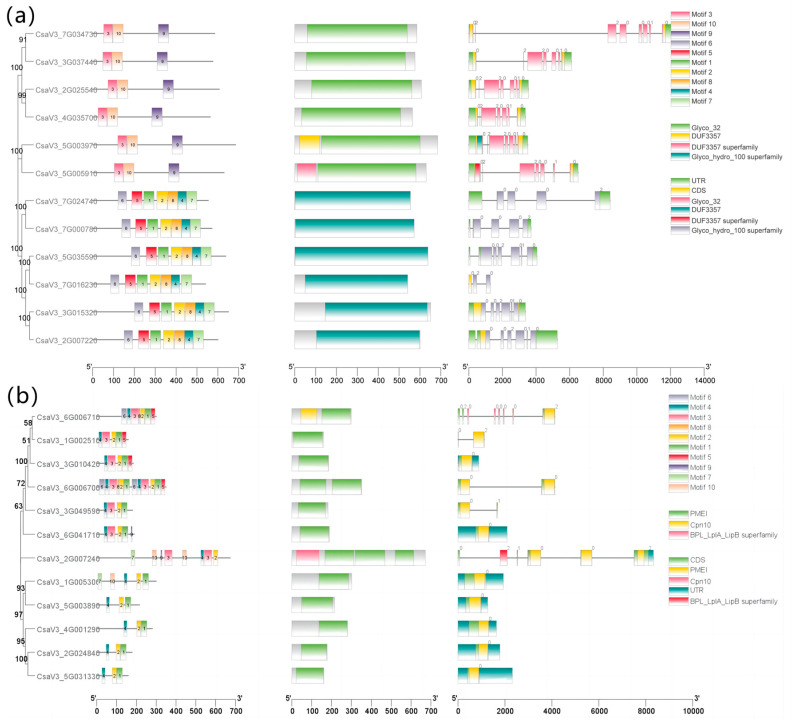
Motif composition and gene structure of *CSINVs* and *CSINHs*. The first column is the phylogenetic tree, followed by the motif, conserved domains, and gene structure. (**a**) Motif composition and gene structure of *CSINVs*; (**b**) motif composition and gene structure of *CSINHs*.

A total of 12 invertase sequences and 12 inhibitor protein sequences were identified from the cucumber genome. The parameters of the gene characteristics, including gene IDs, chromosome location, amino acid length, molecular weight (MW), isoelectric point (pI), instability index, hydropathicity, transmembrane domain, and subcellular localization, were analyzed (Figure 1). The total average negative hydrophilicity of the cucumber invertase and invertase inhibitor protein families accounted for a high proportion, and most of them were unstable proteins. The cucumber invertases and their inhibitor genes were present in the cytoplasm, outer membrane, and periplasm (Supplementary Table S1). According to research on Arabidopsis, invertase gene families are divided into three subfamilies, including cell wall, cytoplasmic, and vacuolar subfamilies. Analysis of conserved protein motifs revealed that the cell wall and vacuolar subfamilies have the same motifs. Moreover, members of the cell wall and vacuolar subfamilies have a typical Glyco_32 domain, and cytoplasmic subfamily members have a Glyco_hydro_100 superfamily domain. Among *CSINHs*, *CsaV3_6G006700* has more motifs than the others, *CsaV3_6G006710* and *CsaV3_2G007240* have unique domains, and they may have functional differentiation. All twelve inhibitors have a typical PMEI domain.

### 2.2. Identification and Characterization of CSINVs and CSINHs

**Figure 2:**
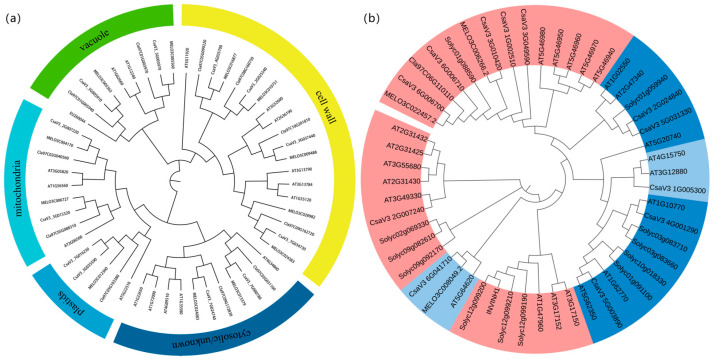
Phylogenetic analysis of *CSINVs* and *CSINHs*. (**a**) Phylogenetic tree of *CSINVs* (cell wall invertase (yellow), vacuolar invertase (green), mitochondrial invertase, plastid invertase, and cytosolic invertase (color from light to dark blue)). (**b**) Phylogenetic tree of *CSINHs*.

To study the evolutionary relationship of cucumber invertase and invertase inhibitor gene families, two phylogenetic trees of 54 invertase genes and 51 invertase inhibitor genes from watermelon, melon, Arabidopsis and tomato were constructed (Figure 2). The results showed that the 12 invertase genes of cucumber were divided into 3 subfamilies, including 4 cell wall, 6 cytoplasmic, and 2 vacuolar subfamilies. Cytoplasmic invertases can be further divided into three subfamilies: mitochondrial invertases, plastid invertases and cytoplasmic invertases, as described in maize [27]. However, the inhibitor gene family has not been divided into cell wall and vacuolar subfamilies, as reported by Wan et al. (2018), which must be further determined experimentally [28].

### 2.3. Chromosomal Location and Collinearity Analysis of CSINVs and CSINHs

**Figure 3:**
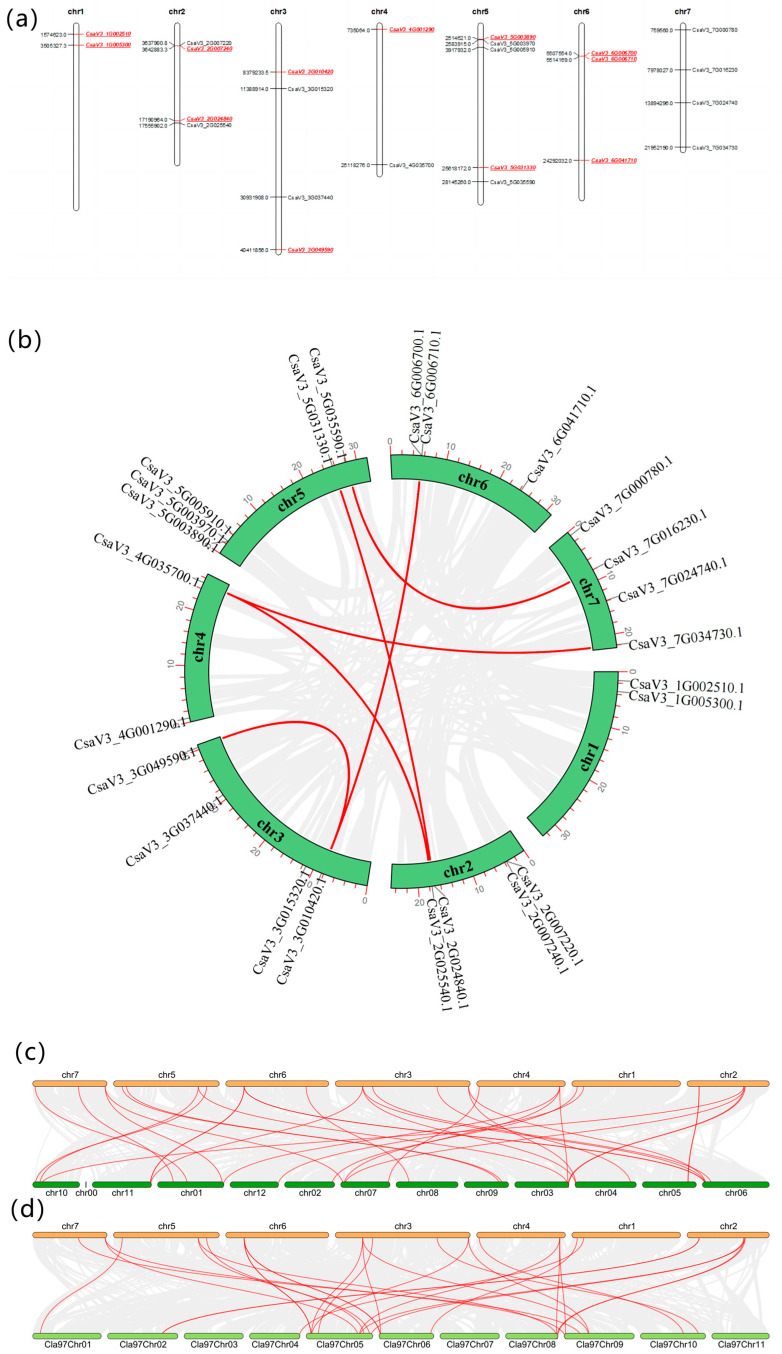
(**a**) Gene location map. Invertase in black, and its inhibitor in red; (**b**) schematic representations of the chromosomal distribution and interchromosomal relationships of *CSINVs* and *CSINHs*. Gray lines in the background indicate all syntenic blocks in the cucumber genome, and red lines indicate segmental duplication gene pairs. (**c**) Synteny analysis of *CSINVs* and *CSINHs* between cucumber and melon. The gray lines indicate all collinear blocks, and the red lines indicate collinear *CSINV* and *CSINH* pairs. (**d**) Synteny analysis of *CSINVs* and *CSINHs* between cucumber and watermelon. The gray lines indicate all collinear blocks, and the red lines indicate collinear *CSINV* and *CSINH* pairs.

Based on the genome sequence information of the relevant chromosome, MapChart was used to map the location of each invertase and inhibitor on the chromosome. Among them, Ch1 has the lowest number (Figure 3a).

By analyzing the segmental duplication events, it was found that there were seven pairs of collinearity genes in cucumber, including six segmental duplication events between different chromosomes that contain *CsaV3_2G025540* and *CsaV3_4G035700*, *CsaV3_2G024840* and *CsaV3_5G031330*, *CsaV3_3G010420* and *CsaV3_6G006700*, *CsaV3_3G010420* and *CsaV3_6G006710*, *CsaV3_4G035700* and *CsaV3_7G034730*, and *CsaV3_5G035590* and *CsaV3_7G016230* and one duplication event within the same chromosome including *CsaV3_3G010420* and *CsaV3_3G049590*, indicating that they were possibly inherited from the same ancestor (Figure 3b). The analysis between melon and cucumber species found that *CsaV3_7G016230* (cytoplasmic invertase) and *CsaV3_5G003890* (invertase inhibitor) have no syntenic genes (Figure 3c). Between watermelon and cucumber, invertases, including *CsaV3_5G005910* (vacuolar invertase), *CsaV3_7G000780* (cytoplasmic invertase), and *CsaV3_7G016230* (cytoplasmic invertase), and the invertase inhibitor *CsaV3_ 5G003890,* all have no syntenic genes (Figure 3d). This suggests that these genes do not have similar sequences and functions and might have been specific to cucumber during evolution. There were slightly more collinear gene pairs between melon and cucumber than watermelon, indicating that cucumber and melon are more closely related than watermelon.

### 2.4. Expression Profiling of CSINVs and CSINHs in Different Tissues and during Growth and Development

**Figure 4:**
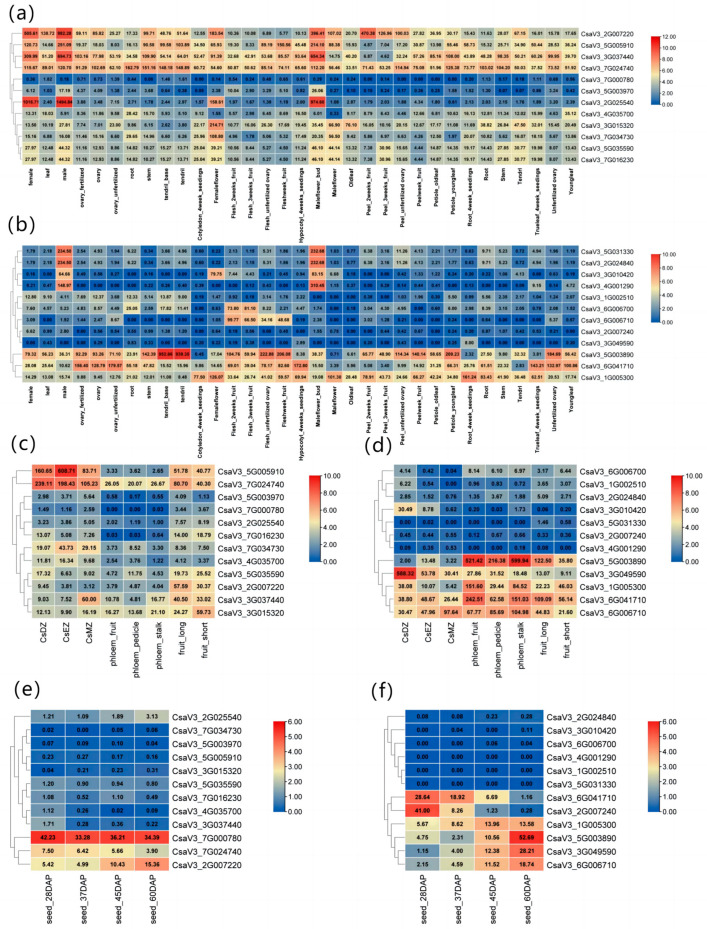
Heatmap of the gene expression of *CSINVs* (**a**,**c**) and *CSINHs* (**b**,**d**) in different cucumber tissues and growth and development stages. CsDZ is the root differentiation zone, CsEZ is the root elongation zone, and CsMZ is the root meristematic zone. Heatmap of gene expression of *CSINVs* (**e**) and *CSINHs* (**f**) in cucumber seeds after pollination. DAP is days after pollination. The data in the boxes represent raw RPKM values.

Using the published RNA-seq data on the CuGenDB website, the expression levels of the twenty-four genes in different cucumber organs or tissues were summarized (Figure 4).

In the root differentiation zone, an invertase gene (*CsaV3_7G024740*) and inhibitor gene (*CsaV3_3G049590*) were highly expressed. In the flowers or flower buds, three invertase genes (*CsaV3_2G025540*, *CsaV3_3G037440,* and *CsaV3_2G007220*) and three inhibitor genes (*CsaV3_5G031330*, *CsaV3_2G024840*, and *CsaV3_4G001290*) were significantly upregulated. In the pollinated cucumber seeds, the expression levels of invertases (*CsaV3_7G000780* and *CsaV3_7G024740*) and inhibitors (*CsaV3_6G041710* and *CsaV3_2G007240*) decreased with time. In contrast, the expression levels of *CsaV3_6G006710* and *CsaV3_3G049590* continued to increase. The expression of *CsaV3_6G041710* and *CsaV3_5G003890* was higher in some young tissues, such as young leaves and petioles of young leaves. However, with the growth of the plants and tissue senescence, their expression decreased.

The expression level of invertase gene *CsaV3_7G024740* and inhibitor genes *CsaV3_6G041710*, *CsaV3_6G006710*, and *CsaV3_5G003890* in the long fruit was twice that in the short fruit; however, the expression level of invertase gene *CsaV3_3G015320* and inhibitor gene *CsaV3_1G005300* was significantly increased in the short fruit.

Invertase genes (*CsaV3_7G024740* and *CsaV3_3G015320*) and inhibitor genes (*CsaV3_6G041710*, *CsaV3_6G006710*, *CsaV3_1G005300*, and *CsaV3_5G003890*) had higher expression in fruit, pedicel, and stalk phloem on the third day after flowering. The invertase gene *CsaV3_3G037440* was highly expressed in stalk phloem, and *CsaV3_5G035590* was highly expressed in the phloem of the pedicel, which may regulate pedicel unloading.

### 2.5. Expression Profile Analysis of CSINVs and CSINHs under Biotic Stress

**Figure 5:**
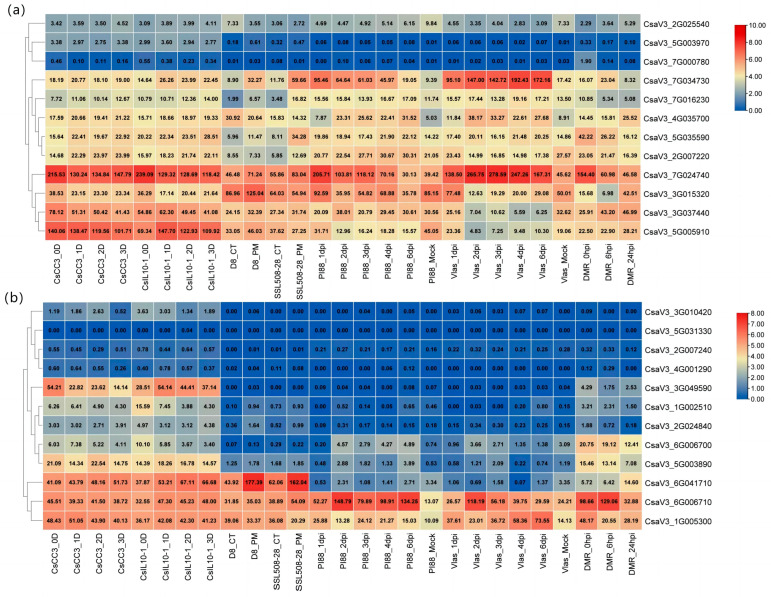
Expression profile of cucumber genes *CSINVs* (**a**) and *CSINHs* (**b**) under biotic stress. CsCC3 refers to nematode-resistant varieties, CsIL10 refers to non-insect-resistant varieties, and the following numbers indicate the days after infection with nematodes. CT refers to mock as a control, PM refers to powdery mildew, D8 is the recurrent parent, and SSL508-28 is the powdery mildew-resistant segment substitution line. PI88 refers to downy mildew-resistant varieties, Vlas is susceptible to downy mildew varieties, and the numbers indicate the days of downy mildew infection. DMR refers to downy mildew resistance, and hpi refers to hours post-infection. The data in the boxes represent raw RPKM values.

To confirm whether invertases are involved in the response to various biotic stresses, the expression patterns of invertases under different biotic stresses were analyzed (Figure 5).

After root-knot nematode infestation, the expression of *CsaV3_7G034730* and *CsaV3_5G005910* significantly increased in non-nematode-resistant varieties. *CsaV3_7G024740* was more downregulated in non-nematode-resistant varieties. Among the invertase inhibitors, the expression of *CsaV3_6G041710* increased in both varieties, but the increase was faster in non-insect-resistant varieties. However, *CsaV3_3G049590* continued to decline in nematode-resistant varieties. In the non-nematode-resistant varieties, the expression level increased rapidly one day after infection with nematodes and then decreased slowly, but the expression level was always higher than that of the nematode-resistant varieties.

Under the stress of powdery mildew inoculation, the expression level of *CsaV3_7G034730* increased more in powdery mildew-resistant varieties. *CsaV3_5G005910*, *CsaV3_2G007220,* and *CsaV3_3G015320* increased in D8 non-resistant powdery mildew-resistant varieties but decreased in disease-resistant varieties. Although *CsaV3_5G035590* and *CsaV3_6G006710* were upregulated in both varieties, the expression level increased threefold in disease-resistant varieties. While *CsaV3_1G005300* was downregulated in both cultivars, it decreased more in the disease-resistant cultivar. These results showed that the above seven cucumber invertase genes were closely related to the powdery mildew resistance of cucumber.

After downy mildew inoculation treatment, the expression of *CsaV3_7G034730* and *CsaV3_7G024740* was downregulated in resistant varieties but significantly upregulated in susceptible varieties. However, *CsaV3_2G025540*, *CsaV3_3G037440*, *CsaV3_4G035700*, *CsaV3_5G005910*, *CsaV3_3G015320,* and *CsaV3_2G007220* were upregulated with time in resistant varieties. After inoculation with downy mildew for three days, the expression level of *CsaV3_6G006710* increased significantly in both resistant and susceptible varieties and then gradually declined. In addition, *CsaV3_1G005300* always maintained a high expression level in susceptible varieties but a low expression level in resistant varieties. Therefore, the abovementioned cucumber genes may be related to the downy mildew resistance of cucumber.

### 2.6. Expression Profiling of CSINVs and CSINHs under Abiotic Stress

**Figure 6:**
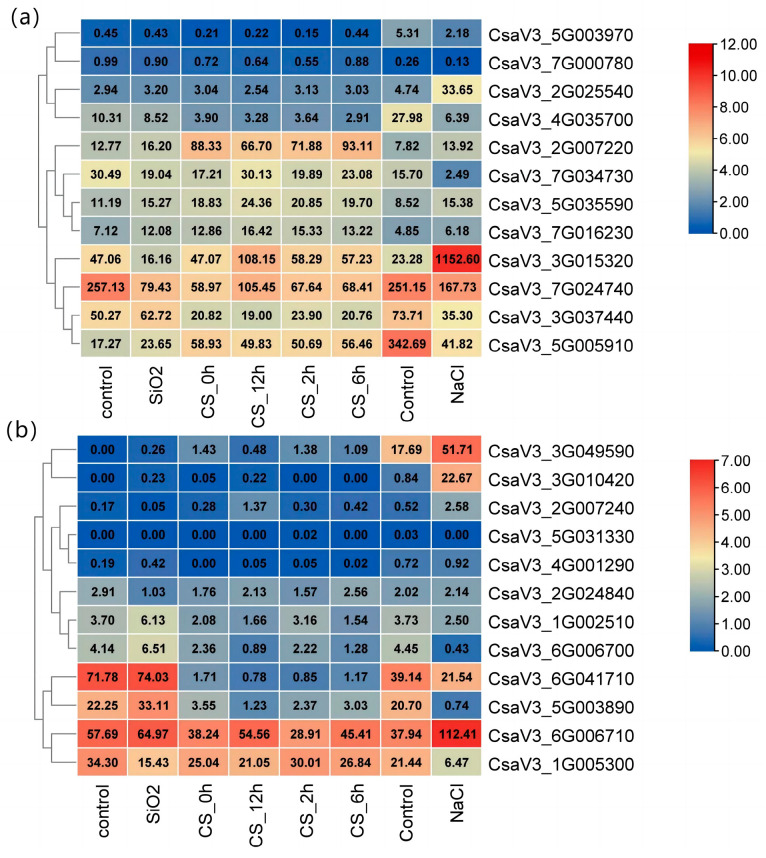
Expression profiles of cucumber genes *CSINVs* (**a**) and *CSINHs* (**b**) under abiotic stress. CS_0h, CS_2h, CS_6h, and CS_12h represent seedlings exposed to 6 °C air temperature for 0, 2, 6, and 12 h, respectively. The data in the box represent the original RPKM values.

Among the invertases, the expression of *CsaV3_5G005910* and *CsaV3_5G003890* was significantly downregulated after treatment with 75 mM NaCl, indicating that these genes are sensitive to salt (Figure 6). However, the expression of *CsaV3_3G015320*, *CsaV3_6G006710,* and *CsaV3_3G049590* was significantly increased after treatment with 75 mM NaCl. After being treated with silicon (SiO_2_), the expression of *CsaV3_7G024740* was significantly downregulated. The expression of *CsaV3_3G015320* was high at low temperature, which may contribute to the anti-low temperature. When the temperature changed, the longer the exposure time of *CsaV3_3G049590* and *CsaV3_6G006700* was at low temperature, the lower the expression level was.

## 3. Discussion

The bioinformatic analysis identified 12 invertase genes and 12 invertase inhibitor genes. The phylogenetic analysis clustered the 12 invertase genes into 3 subfamilies; among them, some orthologous genes from the other three species were functionally identified, which indicated that the cucumber genes in the same subfamilies might involve similar functions. These genes could be preliminarily named four cucumber cell wall invertases (*CsCWIN01-04*), two vacuolar invertases (*CsVIN01-02*), and six cytoplasmic invertases (*CsCIN01-06*) (Supplementary Table S1). However, for inhibitor genes, no subfamily could be divided as previously reported.

In comparison with watermelon and melon, most cucumber invertase and invertase inhibitor genes were conserved; however, two genes, *CsaV3_7G016230* (*CsCIN04*) and *CsaV3_5G003890* (*CSINH12*), did not have orthologues in melon, and four genes, *CsaV3_5G005910* (*CsVIN02*), *CsaV3_7G000780* (*CsCIN02*), *CsaV3_7G016230* (*CsCIN04*), and *CsaV3_5G003890* (*CSINH12*), did not have orthologues in watermelon. Among them, two genes, *CsaV3_7G016230* (*CsCIN04*) and *CsaV3_5G003890* (*CSINH12*), were unique to cucumber. These genes might have peculiar functions in cucumber. The transcriptome analysis revealed that the downregulation of *CsaV3_5G005910* (*CsVIN02*) might be involved in cucumber resistance to nematodes, powdery mildew, downy mildew, and salinity.

Based on the phylogenetic trees, potential functions of cucumber invertase and its inhibitor genes were deduced in Supplementary Table S2.

The cucumber gene *CsaV3_2G025540* (*CsCWIN02*) has two duplicate orthologues both in melon (*MELO3C010751* and *MELO3C016877*) and watermelon (*Cla97C05G099220* and *Cla97C08G160750*), where *MELO3C010751* [29] and *Cla97C05G099220* [30] were reported to be highly expressed during fruit development, which indicated that *CsCWIN02* might share a similar function in cucumber and may be beneficial for regulating fruit development and taste quality, which needs further study.

The expression of six invertases and inhibitors in cucumber was verified by qRT–PCR. As shown in Supplementary Figure S1, *CsaV3_7G024740* was significantly upregulated in the young fruits, *CsaV3_4G001290* was significantly upregulated in the male flowers, and *CsaV3_5G005910* was significantly downregulated in the roots, which was consistent with the transcriptome patterns. The other three genes did not show significant variations in different tissues, which is inconsistent with the transcriptome patterns, which we speculate is due to the different cucumber varieties, site, and period of sampling.

*CsaV3_7G024740* (*CsCIN01*) and *CsaV3_7G000780* (*CsCIN02*) might correspond to the young fruit growth of cucumber as *MELO3C024083.2*. Moreover, *CsaV3_6G041710* (*CSINH02*) and *CsaV3_3G010420* (*CSINH05*) were clustered with *MELO3C006266.2* and *MELO3C008049.2* into two groups, respectively. *CSINH02* and *CSINH05* may affect juvenile fruit development, similar to *MELO3C006266.2* and *MELO3C008049.2* [31].

*CsaV3_3G015320* (*CsCIN05*) was clustered with *MELO3C006727* in the same group. Since the expression of *MELO3C006727* increased during ripening and peaked in the mature fruit [31], *CsCIN05* may play an important role in promoting fruit development in cucumber.

*CsaV3_5G035590* (*CsCIN03*) and *CsaV3_7G016230* (*CsCIN04*) were clustered with *MELO3C012360*, which played a major role in sucrose accumulation [32]. It can be predicted that *CsCIN03* and *CsCIN04* may have the same function.

*CsaV3_3G049590* may promote endosperm cellularization embryonic growth, similar to *AT5G46950* and *AT5G46960* [33].

*CsaV3_4G001290* (*CSINH10*) and *CsaV3_1G005300* (*CSINH11*) were clustered with *AT1G10770*, which could make males fertile, promote pollen tube growth, and increase the rate of fruit [34]. *CSINH10* and *CSINH11* may share the same abilities.

## 4. Materials and Methods

### 4.1. Plant Materials and Treatments

Seeds of cucumber (*C. sativus* L. “Jinchun 5”) were sown in a plastic bowl of 10 × 10 cm with a 2:1 substrate ratio of peat:vermiculite (*V*/*V*). The plastic bowl was placed in an artificial climate chamber under a photoperiod of 12 h light a day (6:00 to 18:00, Philip HPLN 400 W, 700 µmol/(m^2^·s)), and the relative humidity was controlled at approximately 70%. Once the third true leaves appeared, the seedlings were transferred to a greenhouse. Then, the seedlings were watered every 2 days by a drip irrigation belt. For RT–PCR, different organs were taken when the plants had grown to 15 leaves. Samples of female and male flowers were taken on the day of flowering. The roots were intact as they were being sampled. Samples of fruit were taken seven days after flowering.

### 4.2. Identification and Characterization of CSINVs and CSINHs

Fifty-three known invertase protein sequences and forty-eight known invertase inhibitor protein sequences from *Citrullus lanatus* (watermelon), *Cucumis melo* (melon), *Arabidopsis thaliana* (Arabidopsis), and *Solanum lycopersicum* (tomato), which were collected from the NCBI database (http://www.ncbi.nlm.nih.gov/, accessed on 28 November 2022) and Tair (https://www.arabidopsis.org/, accessed on 28 November 2022), were used as query sequences. Twelve invertase sequences and 12 invertase inhibitor sequences were identified in the cucumber genome downloaded from the CuGenDB website (http://cucurbitgenomics.org/, accessed on 28 November 2022) [35]. The conserved domains of the candidate genes were verified using the NCBI database (http://www.ncbi.nlm.nih.gov/, accessed on 28 September 2022) [36]. The physicochemical properties of cucumber invertase and inhibitor gene sequences, including amino acid composition, relative MW, and pI, were analyzed by ExPASy-Protparam online software (https://web.expasy.org/compute_pi/, accessed on 5 June 2022) [37]; SignalP 3.0 (https://services.healthtech.dtu.dk/service.php?SignalP-3.0, accessed on 5 June 2022) was used to predict signal peptides.

### 4.3. Motif Composition and Gene Structural Analysis of CSINVs and CSINHs

Conserved motifs of proteins were analyzed using the online site MEME (http://meme-suite.org/, accessed on 27 January 2022) [38]. The structural domain was predicted with the Hitdata file obtained by the NCBI (http://www.ncbi.nlm.nih.gov/, accessed on 27 January 2022), and the gene structure was analyzed with the gff3 file visualized by TBtools v1.09876 (accessed on 27 January 2022) [39]. The gene family promoter sequences containing 1000 bp DNA from the cucumber whole genome were extracted by TBtools v1.09876 (accessed on 27 January 2022). The plant cis-acting regulatory element PlantCARE website (http://bioinformatics.psb.ugent.be/webtools/plantcare/html/, accessed on 27 January 2022) was applied to predict the cucumber promoter cis-acting elements, and TBtools was used for visual analysis v1.09876 (accessed on 27 January 2022) [40].

### 4.4. Phylogenetic Tree Construction of CSINVs and CSINHs

Putative cucumber invertase and invertase inhibitors and functional invertase and invertase inhibitors in other species (watermelon, melon, Arabidopsis, and tomato) were used to perform the phylogenetic analysis. Invertases were aligned by full sequence by Clustal X (1.83) (Do complete alignment) [41], then constructed by the wag model of FastTree (accessed on 3 February 2023) [42], visualized by FigTree v1.4.3, and modified with Adobe Illustrator CS5.1. Invertase inhibitor phylogenetic and molecular evolutionary analyses were conducted based on the neighbor joining method of MEGA version 7. Trees constructed with 2000 bootstrap replicates yielded identical clustering.

### 4.5. Analysis of Chromosomal Location and Orthologues of CSINVs and CSINHs

Based on the genome sequence information of the relevant chromosome, MapChart (accessed on 28 February 2023) was used to map the location of each invertase and invertase inhibitor on the chromosome. Gene duplication events were detected by MCScanX [43]. Synteny relationships of the orthologues within cucumber, between cucumber and melon, and between cucumber and watermelon were exhibited by TBtools software v1.09876 (accessed on 28 February 2023) [44].

### 4.6. Differential Gene Expression Profiles Based on RNA-seq of CSINVs and CSINHs

To investigate the expression profiles of invertase genes among different organs and developmental stages, RNA-seq data from various tissues in *C. sativus* were downloaded from the CuGenDB website (http://cucurbitgenomics.org/, accessed on 27 January 2022). Normalized gene expression values were estimated by fragments per kilobase pair of exon model per million fragments mapped (FPKM). Finally, log2-transformed FPKM values of invertase genes in these tissues were used to draw heatmaps. To avoid taking the log of a number less than 1, all such FPKM values were replaced by 1. The expression data were hierarchically clustered using TBtools software v1.09876 (accessed on 2 March 2022) [45].

## Data Availability

Not applicable.

## Supplementary Materials

The supporting information can be downloaded at: https://www.mdpi.com/article/10.3390/ijms241713421/s1. References [46,47,48,49,50,51,52,53,54,55,56,57,58,59,60,61,62,63,64,65] is cited in Supplementary Materials.
