# Supplementary material for: Genome-Wide Identification and Expression Patterns of Cucumber Invertases and Their Inhibitor Genes
Source: Int J Mol Sci. 2023 Aug 30;24(17):13421. doi: 10.3390/ijms241713421 (PMC10487868; doi:10.3390/ijms241713421)
Supplement: Supplementary file 1 [file ijms-24-13421-s001.zip › Figure S1 Transcript levels of invertase members were measured from female flowers, male flowers, roots, and young fruits.+Table S3 qRT-PCR primers for candidate gene validation..pdf]

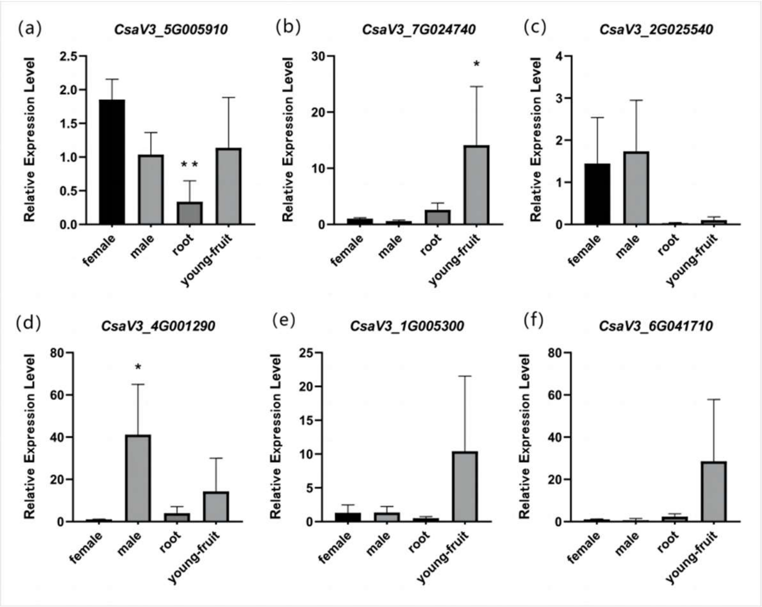


**Figure S1:** Transcript levels of invertase members were measured from female flowers, male flowers, roots, and young fruits. Results are means of =3 biological replicates (±SE) each with three technical replicates. Asterisks indicate statistically significant differences (*, p-value <0.05; **, p-value <0.05).

## Methods

*RNA extraction and qRT-PCR*

Male flowers, female flowers, roots and fruits were taken and preserved in the -80°C refrigerator. An RNAiso Plus kit (TaKaRa, Dalian, China) was used to isolate the total RNA from various tissues of cucumber. The primer design was performed using Primer 3 Plus (http://www.primer3plus.com/). All primers (Table 1) were designed for isoform specificity and optimized for amplicon size = 70–150 bp, GC content=40–60%, and annealing temperature = 55–60 °C. A real-time PCR analysis (qRT–PCR) was performed using a One Step SYBR PrimeScript RT–PCR kit (TaKaRa, Dalian, China) with the CFX96 Touch™ Real-Time PCR Detection System (Bio-Rad, Berkeley, CA, USA). The cycling protocol was as follows: 3 min at 95 °C, 39 cycles of 10 s at 95 °C, 30 s at 50 °C, and a melting curve program (65-95 °C, with a heating rate of 0.5 °C per second). Normalization was performed using the 18S rRNA gene (*Csa2G252100*) (Supplementary Table S3).

**Table S3:** qRT-PCR primers for candidate gene validation.

| Gene ID | Forward primers (5'-3') | Reverse primers (5'-3') |
|---|---|---|
| *CsaV3_5G005910* | GACAGGCAGCAACATAATCC | TCGAGGAGCACGTCATTAAA |
| *CsaV3_7G024740* | AGTCGGTTGCTGAAGGATAG | TCCTCGCTTGTTTTCCGATA |
| *CsaV3_2G025540* | AGGTTTCCTTTGAGCTTCCA | TCTATGTCTACAAACGCCCC |
| *CsaV3_4G001290* | GAAACATCGCCTTCTCAACC | CCAAACCTCCACCAAGTAGT |
| *CsaV3_1G005300* | AGTCTGATCGTCGGAGTCTA | TGTGGGCAGCGTTATTAGAT |
| *CsaV3_6G041710* | ATTTCCATGGACTTGTTGCG | CAATGTCGGAACAGCATCAG |
