# Supplementary material for: Genome-Wide Identification and Expression Patterns of Cucumber Invertases and Their Inhibitor Genes
Source: Int J Mol Sci. 2023 Aug 30;24(17):13421. doi: 10.3390/ijms241713421 (PMC10487868; doi:10.3390/ijms241713421)
Supplement: Supplementary file 1 [file ijms-24-13421-s001.zip › References of Table S2.pdf]

References

1. Cheng, J.T.; Li, X.; Yao, F.Z.; Shan, N.; Li, Y.H.; Zhang, Z.X.; Sui, X.L. Functional characterization and expression analysis of cucumber (Cucumis sativus L.) hexose transporters, involving carbohydrate partitioning and phloem unloading in sink tissues. *Plant Sci* **2015**, *237*, 46-56, doi:10.1016/j.plantsci.2015.05.006.
2. Zhang, H.; Wang, H.; Yi, H.; Zhai, W.; Wang, G.; Fu, Q. Transcriptome profiling of Cucumis melo fruit development and ripening. *Horticulture Research* **2016**, *3,* 16014, doi:10.1038/hortres.2016.14.
3. Veillet, F.; Gaillard, C.; Coutos-Thévenot, P.; La Camera, S. Targeting the AtCWIN1 Gene to Explore the Role of Invertases in Sucrose Transport in Roots and during Botrytis cinerea Infection. *Front Plant Sci* **2016**, *7*, 1899, doi:10.3389/fpls.2016.01899.
4. Tymowska-Lalanne, Z.; Kreis, M. Expression of the Arabidopsis thaliana invertase gene family. *Planta* **1998**, *207*, 259-265, doi:10.1007/s004250050481.
5. Guo, S.; Sun, H.; Zhang, H.; Liu, J.; Ren, Y.; Gong, G.; Jiao, C.; Zheng, Y.; Yang, W.; Fei, Z.; et al. Comparative Transcriptome Analysis of Cultivated and Wild Watermelon during Fruit Development. *Plos One* **2015**, *10*, e0130267, doi:10.1371/journal.pone.0130267.
6. Wu, P.; Xiao, C.; Cui, J.; Hao, B.; Zhang, W.; Yang, Z.; Ahammed, G.J.; Liu, H.; Cui, H. Nitric Oxide and Its Interaction with Hydrogen Peroxide Enhance Plant Tolerance to Low Temperatures by Improving the Efficiency of the Calvin Cycle and the Ascorbate–Glutathione Cycle in Cucumber Seedlings. *Journal of Plant Growth Regulation* **2020**, *40*, 2390-2408, https://doi.org/10.1007/s00344-020-10242-w.7. Liao, S.; Wang, L.; Li, J.; Ruan, Y.L. Cell Wall Invertase Is Essential for Ovule Development through Sugar Signaling Rather Than Provision of Carbon Nutrients. *Plant Physiol* **2020**, *183*, 1126-1144, doi:10.1104/pp.20.00400.
8. Gao, L.; Zhao, S.; Lu, X.; He, N.; Zhu, H.; Dou, J.; Liu, W. Comparative transcriptome analysis reveals key genes potentially related to soluble sugar and organic acid accumulation in watermelon. *PLOS ONE* **2018**, *13*, e0190096, doi:10.1371/journal.pone.0190096.
9. Zhu, Q.; Gao, P.; Liu, S.; Zhu, Z.; Amanullah, S.; Davis, A.R.; Luan, F. Comparative transcriptome analysis of two contrasting watermelon genotypes during fruit development and ripening. *BMC Genomics* **2017**, *18*, 3, doi:10.1186/s12864-016-3442-3.
10. Wang, J.; Wang, Y.; Zhang, J.; Ren, Y.; Li, M.; Tian, S.; Yu, Y.; Zuo, Y.; Gong, G.; Zhang, H.; et al. The NAC transcription factor ClNAC68 positively regulates sugar content and seed development in watermelon by repressing ClINV and ClGH3.6. *Hortic Res* **2021**, *8*, 214, doi:10.1038/s41438-021-00649-1.
11. Feng, Z.; Zheng, F.; Wu, S.; Li, R.; Li, Y.; Zhong, J.; Zhao, H. Functional Characterization of a Cucumber (Cucumis sativus L.) Vacuolar Invertase, CsVI1, Involved in Hexose Accumulation and Response to Low Temperature Stress. *Int J Mol Sci* **2021**, *22*, 9365, doi:10.3390/ijms22179365.
12. Wu, Z.; Tu, M.; Yang, X.; Xu, J.; Yu, Z. Effect of cutting and storage temperature on sucrose and organic acids metabolism in postharvest melon fruit. *Postharvest Biology and Technology* **2020**, *161*, 111081, doi:https://doi.org/10.1016/j.postharvbio.2019.111081.
13. Schemberger, M.O.; Stroka, M.A.; Reis, L.; de Souza Los, K.K.; de Araujo, G.A.T.; Sfeir, M.Z.T.; Galvão, C.W.; Etto, R.M.; Baptistão, A.R.G.; Ayub, R.A. Transcriptome profiling of non-

climacteric 'yellow' melon during ripening: insights on sugar metabolism. *BMC Genomics* **2020**, *21*, 262, doi:10.1186/s12864-020-6667-0.
14. Tian, H.; Kong, Q.; Feng, Y.; Yu, X. Cloning and characterization of a soluble acid invertase-encoding gene from muskmelon. *Mol Biol Rep* **2009**, *36*, 611-617, doi:10.1007/s11033-008-9219-2.
15. Patzke, K.; Prananingrum, P.; Klemens, P.A.W.; Trentmann, O.; Rodrigues, C.M.; Keller, I.; Fernie, A.R.; Geigenberger, P.; Bölter, B.; Lehmann, M.; et al. The Plastidic Sugar Transporter pSuT Influences Flowering and Affects Cold Responses. *Plant Physiol* **2019**, *179*, 569-587, doi:10.1104/pp.18.01036.
16. Wang, J.; Sun, W.; Kong, X.; Zhao, C.; Li, J.; Chen, Y.; Gao, Z.; Zuo, K. The peptidyl-prolyl isomerases FKBP15-1 and FKBP15-2 negatively affect lateral root development by repressing the vacuolar invertase VIN2 in Arabidopsis. *Planta* **2020**, *252*, 52, doi:10.1007/s00425-020-03459-2.
17. Pignocchi, C.; Ivakov, A.; Feil, R.; Trick, M.; Pike, M.; Wang, T.L.; Lunn, J.E.; Smith, A.M. Restriction of cytosolic sucrose hydrolysis profoundly alters development, metabolism, and gene expression in Arabidopsis roots. *Journal of Experimental Botany* **2021**, *72*, 1850-1863, doi:10.1093/jxb/eraa581.
18. Yang, J.; Deng, G.; Lian, J.; Garraway, J.; Niu, Y.; Hu, Z.; Yu, J.; Zhang, M. The Chromosome-Scale Genome of Melon Dissects Genetic Architecture of Important Agronomic Traits. *iScience* **2020**, *23*, 101422, doi:10.1016/j.isci.2020.101422.
19. Maruta, T.; Otori, K.; Tabuchi, T.; Tanabe, N.; Tamoi, M.; Shigeoka, S. New insights into the regulation of greening and carbon-nitrogen balance by sugar metabolism through a plastidic invertase. *Plant Signal Behav* **2010**, *5*, 1131-1133, doi:10.4161/psb.5.9.12568.
20. Battaglia, M.E.; Martin, M.V.; Lechner, L.; Martínez-Noël, G.M.A.; Salerno, G.L. The riddle of mitochondrial alkaline/neutral invertases: A novel Arabidopsis isoform mainly present in reproductive tissues and involved in root ROS production. *PLoS One* **2017**, *12*, e0185286, doi:10.1371/journal.pone.0185286.
21. Zuma, B.; Dana, M.B.; Wang, D. Prolonged Expression of a Putative Invertase Inhibitor in Micropylar Endosperm Suppressed Embryo Growth in Arabidopsis. *Front Plant Sci* **2018**, *9*, 61, doi:10.3389/fpls.2018.00061.
22. Zhang, G.Y.; Feng, J.; Wu, J.; Wang, X.W. BoPMEI1, a pollen-specific pectin methylesterase inhibitor, has an essential role in pollen tube growth. *Planta* **2010**, *231*, 1323-1334, doi:10.1007/s00425-010-1136-7.
23. Liu, T.; Yu, H.; Xiong, X.; Yue, X.; Yu, Y.; Huang, L.; Cao, J. Genome-Wide Identification, Molecular Evolution, and Expression Profiling Analysis of Pectin Methylesterase Inhibitor Genes in Brassica campestris ssp. chinensis. *Int J Mol Sci* **2018**, *19*, 1338, doi:10.3390/ijms19051338.
24. Xu, X.X.; Hu, Q.; Yang, W.N.; Jin, Y. The roles of call wall invertase inhibitor in regulating chilling tolerance in tomato. *BMC Plant Biol* **2017**, *17*, 195, doi:10.1186/s12870-017-1145-9.
25. Su, T.; Wolf, S.; Han, M.; Zhao, H.; Wei, H.; Greiner, S.; Rausch, T. Reassessment of an Arabidopsis cell wall invertase inhibitor AtCIF1 reveals its role in seed germination and early seedling growth. *Plant Mol Biol* **2016**, *90*, 137-155, doi:10.1007/s11103-015-0402-2.
